# Violence suffered by nurses in risk classification and suspicion of common mental disorders

**DOI:** 10.1590/0034-7167-2024-0087

**Published:** 2025-06-20

**Authors:** Daniel da Silva Granadeiro, Raquel Magalhães de Azeredo Granadeiro, Harlon França de Menezes, Renata da Silva Hanzelmann, Giovana Cópio Vieira, Juliane Ferreira da Silva, Maria Isabel Silva Santos, Joanir Pereira Passos

**Affiliations:** I Universidade do Estado do Rio de Janeiro. Rio de Janeiro, Rio de Janeiro, Brazil; II Centro Universitário Augusto Motta. Rio de Janeiro, Rio de Janeiro, Brazil; III Hospital Pró-Cardíaco. Rio de Janeiro, Rio de Janeiro, Brazil; IV Faculdade São José. Rio de Janeiro, Rio de Janeiro, Brazil; V Universidade Federal do Estado do Rio de Janeiro. Rio de Janeiro, Rio de Janeiro, Brazil

**Keywords:** Violence at Work, Mental Disorders, Work Conditions, Sorting, Nurses., Violencia Laboral, Trastornos Mentales, Condiciones de Trabajo, Triaje, Enfermeras y Enfermeros.

## Abstract

**Objective:**

To analyze the association between violence suffered by nurses in the reception service with risk classification and the suspicion of common mental disorders.

**Methods:**

Cross-sectional study, conducted in the city of Rio de Janeiro, with 56 nurses in the reception service with risk classification in the emergency and urgency network. The Questionnaire for Assessment of Violence at Work and the Self-Report Questionnaire were used. The Wilcoxon, chi-square, and Fisher’s exact tests were used for analysis.

**Results:**

The threat variable had a statistically significant difference (p = 0.031). Among the grouped symptoms, the predominant complaint of factor 1 (depressed/anxious mood) in the question “feeling nervous, tense, or worried” had 42 (75%) positive answers.

**Conclusion:**

The violence in the reception service suffered by nurses reflects in the suspicion of mental disorders, mainly related to the environment, abandonment, and depressive symptoms.

## RESUMEN

**Objetivo:**

Analizar la asociación entre la violencia sufrida por enfermeras en el servicio de recepción con la clasificación de riesgo y la sospecha de trastornos mentales comunes.

**Métodos:**

Estudio transversal, realizado en el Municipio de Río de Janeiro, con 56 enfermeros de acogida con clasificación de riesgo de la red de urgencia y emergencia. Se utilizó el Cuestionario de Evaluación de la Violencia en el Trabajo y el Cuestionario de Autoinforme. Para el análisis se utilizaron las pruebas de Wilcoxon, chi-cuadrado y exacta de Fisher.

**Resultados:**

La variable amenaza tuvo diferencia estadísticamente significativa (p= 0,031). Entre los síntomas agrupados, la queja predominante del factor 1 (estado de ánimo depresivo/ansioso) en la pregunta “sentirse nervioso, tenso o preocupado” tuvo 42 (75%) respuestas positivas.

**Conclusión:**

La violencia en recepción sufrida por enfermeras refleja la sospecha de trastornos mentales, principalmente relacionados con el entorno, abandono y síntomas depresivos.

## INTRODUCTION

Workplace violence is defined as any action, incident, or behavior that deviates from what is considered normal. It involves situations where an individual is attacked, threatened, humiliated, or harmed by another person while performing their professional duties^(1)^. This phenomenon, particularly prevalent among nurses in the healthcare field, is widely recognized by international health organizations, with prevalence rates ranging from 17% to 94%. Additionally, it is reported that 8% to 38% of healthcare professionals have experienced violence at some point in their careers ^(2-4)^.

The work process of nurses in emergency services is based on seeking support, through the risk classification tool, and proposing solutions, in an agile and timely manner. Risk classification aims to assess and identify the needs for priority care, according to clinical severity, potential risk, health problems or degree of suffering, being the first care action^(4)^. Therefore, the nurse who classifies must rely on clinical reasoning through methods specific to his/her knowledge, adopt an immediate classification and guide his/her actions with a view to humanizing care.

Emergency care settings are inherently dynamic, involving structured processes to ensure patient care. Studies indicate that the waiting time between initial registration and risk classification may vary depending on current demand, potentially leading to prolonged wait times and delays in treatment initiation ^(5)^. Such delays can create opportunities for violent incidents to arise.

Within this context, the risk classification phase can be particularly stressful, often resulting in overwork and heightened tension, which negatively impact healthcare professionals’ physical and mental health. Workplace stressors serve as critical determinants of professional health outcomes and may contribute to the development of Common Mental Disorders (CMDs). CMDs are characterized by non-psychotic symptoms associated with stress, anxiety, and depression, which can disrupt bodily functions and lead to issues such as insomnia, irritability, fatigue, difficulty concentrating, forgetfulness, and somatic complaints^(6,7)^.

The presence of CMDs has an impact on the quality of care and patient safety, on relationships with the work team, family and society, on job satisfaction, on changes in the health of professionals and absenteeism, in addition to generating additional costs for institutions. Health care can directly affect professionals in this area, bringing changes in quality of life, organizational indicators, as well as in the production of care^(8,9)^.

To gain a comprehensive understanding of workplace violence in nursing, its contexts, and its correlation with workers’ health, it is essential to examine the relationship between healthcare professionals and their work, alongside their mental well-being. Although violence against nurses has been a subject of research in Brazil and worldwide, significant gaps remain when it comes to studying this phenomenon in triage and risk classification (TRC) services, particularly its association with CMDs. While reviewing academic research to explore this phenomenon and its context, it became evident that the topic has not been adequately addressed.

Understanding the influence of work organization on quality of life, mental health, the generation of psychological suffering, exhaustion and illness among workers is of fundamental importance not only for understanding and intervening in work situations that may lead to various forms of suffering, but for overcoming and transforming these work organizations.

## OBJECTIVE

To analyze the association between violence experienced by nurses working in triage and risk classification services and the suspicion CMDs.

## METHODS

### Ethical Aspects

This study was approved by the Ethics Committee of the proposing institution and by the other institutions involved as co-participants, adhering to the ethical principles for research as outlined in Resolution No. 466/2012 of the National Health Council.

### Study Design, Period, and Location

This cross-sectional study was guided by the Strengthening the Reporting of Observational Studies in Epidemiology (STROBE) tool and conducted in Rio de Janeiro, Brazil. Data were collected from October to December 2021 in the risk classification units of general hospitals, Regional Emergency Coordination Centers (CER in Portuguese), and Emergency Care Units (UPA in Portuguese) within the municipal emergency care network of the Municipal Health Department (SMS in Portuguese).

Currently, the SMS manages 29 emergency care units across five programmatic areas. These settings were selected due to their high coverage within the state’s public healthcare network (SUS) and their elevated risk for workplace violence.

Patient care in these units begins with TRC. The facilities are equipped to provide comprehensive treatment and recovery for patients with clinical and traumatic conditions, including hospitalization, surgery, and intensive care services.

### Population, Inclusion, and Exclusion Criteria

The study population consisted of nurses working in the risk classification units. The sample was selected non-probabilistically using the Snowball sampling method, whereby participants were recruited through referral chains ^(10)^.

Inclusion criteria for participants included: actively working as a nurse during the data collection period, regardless of employment type; having at least 12 months of work experience in triage services; and having access to email or the WhatsApp Messenger® mobile application on their phone. These professionals worked 30-hour weekly shifts, covering both day and night schedules.

The length of service was determined because it is understood that this period is essential with regard to experiences related to the work environment and to standardize the minimum time required to use the scale. In turn, nurses without employment ties to the services (interns, residents, preceptors and academics), professionals absent due to vacation, sick leave, pregnancy leave and/or medical certificate and questionnaires that presented less than 50% of completed responses were excluded.

### Study Protocol

Data collection began with five referral chains corresponding to hospital units within each AP. Due to COVID-19-related measures in effect at the time, data were collected electronically.

After contacting professionals from each unit, appointments were scheduled to obtain their email addresses or WhatsApp® contact information for sending the Google Forms® survey link. The Google Forms® platform was divided into four sections: the Informed Consent Form (ICF), Sociodemographic Questionnaire, Workplace Violence Assessment Questionnaire, and Self-Report Questionnaire (SRQ-20).

Once the data collection period ended, the survey link was deactivated, and the collected data were exported from Google Forms® and stored locally using Microsoft Excel®. The online ICF served as the initial survey page, containing information about the study and requesting consent for data usage. Participants could only access the self-administered questionnaires after providing electronic consent.

The Sociodemographic Questionnaire consisted of 21 openand closed-ended questions designed to characterize study participants.

The Workplace Violence Assessment Questionnaire, adapted from the American Federation of State, County, and Municipal Employees ^(11)^, was based on the *Survey Questionnaire Workplace Violence in the Health Sector* developed by the World Health Organization (WHO) and the International Labour Organization (ILO). Adjustments were made to tailor the instrument to the risk classification context and the nursing population, as the original questionnaire was intended for all healthcare professionals. The final instrument consisted of five sections with 46 questions: workplace safety conditions (3 questions), workplace violence training (2 questions), workplace violence prevention policies (5 questions), workplace violence episodes (29 questions), and personal opinions (8 questions).

The Self-Report Questionnaire (SRQ), recommended by WHO and validated in Brazil since 1986, comprises 20 easily applicable “yes” or “no” questions about physical and psychological symptoms experienced in the last 30 days. It evaluates psychosomatic symptoms (e.g., headaches, indigestion, and unpleasant stomach sensations), depressive symptoms (e.g., sadness, frequent crying, and loss of interest), and anxiety symptoms (e.g., poor sleep, startling easily, tremors, and nervousness) ^(9)^.

The SRQ is a reliable tool for evaluating CMDs, as it effectively identifies factors essential for occupational mental health screening. The cut-off score for CMD suspicion in this study was 7 for both sexes, based on validation studies indicating optimal sensitivity and specificity ^(12)^.

### Data Analysis and Statistics

Data analysis included descriptive statistics presented in tables, with absolute (n) and relative (%) frequencies, means, standard deviations, medians, and minimum and maximum values.

Descriptive analysis involved measures of central tendency and frequency distributions for dichotomous qualitative variables, estimating prevalence and assessing association significance. A binary logistic regression model was used to analyze risk factors for CMDs, incorporating sociodemographic, occupational, and violence-related predictors.

Inferential analysis utilized the Wilcoxon test for numerical outcome variables. For categorical variables, the chi-square test was applied if all contingency table substrata contained more than five observations; otherwise, Fisher’s exact test was used. A significance level of 5% was adopted. Statistical analyses were performed using R software, version 4.0.

## RESULTS

The final sample consisted of 56 nurses, predominantly female (n=41; 73%), White (n=25; 45%), single (n=32; 57%), and aged between 34 and 45 years. Regarding education, 48 participants (86%) had specialized training, but 33 (59%) had not received any formal training to perform their professional duties in TRC services.

Table 1 presents the distribution of occupational characteristics among nurses working in TRC.

**Table 1:** Distribution of study participants by occupational characteristics, Rio de Janeiro, RJ, Brazil, 2022

Variables	*F*	%
Area of the city of Rio de Janeiro where you work		
Center	9	16
North Zone	23	41
West Zone	16	29
South Zone	5	9
I don't know	3	5
Workplace^*^		
UPA	31	55
CER	10	18
General Hospital	19	34
Has received training to work at TRC		
No	33	59
Yes	23	41
Shift of work at TRC		
Daytime Service	12	21
Night Service	4	7
Both	40	72
Employment Contract^*^		
Statutory	5	9
CLT	39	70
Outsourced (OSS)	17	30
Total working hours at TRC		
Greater than 30 hours	17	30
Less than or = 30 hours	39	70
Number of employment contracts		
1	24	43
2 or more	32	57
Knowledge about violence in the workplace		
No	1	2
Yes	54	96
I don't know	1	2

*Notes: Participants could select more than one option in this variable. UPA (Emergency Care Unit); CER (Regional Emergency Coordination); TRC (Triage and Risk Classification); CLT (Consolidation of Labor Laws); OSS (Social Health Organizations).*

Regarding variables such as the area of Rio de Janeiro where the nurse worked, training for triage duties, length of professional activity, shift and working hours, number of employment contracts, total weekly working hours, and overall length of professional experience, no statistically significant differences (p<0.05) were observed in association with suspected CMDs (Table 2).

**Table 2:** Suspected CMDs in the sample and their association with occupational characteristics, Rio de Janeiro, RJ, Brazil, 2022

Variables	Suspected CMDs		*p* value*^2^*
	Cutoff point < 7	Cutoff point > 7	
	n30*^1^*	n26*^1^*	
Workplace			
UPA			
No	17 (57%)	8 (31%)	0.052
Yes	13 (43%)	18 (69%)	
CER			
No	24 (80%)	22 (85%)	0.7
Yes	6 (20%)	4 (15%)	
General Hospital			
No	17 (57%)	20 (77%)	0.11
Yes	13 (43%)	6 (23%)	

Notes: Common Mental Disorders (CMDs); Emergency Care Unit (UPA); Regional Emergency Coordination (CER);

1 Median (IQR);

2 Chi-Square

Table 3 displays the associations between variables such as abandonment or transfer from triage and risk classification services due to experiencing or witnessing episodes of violence and suspected CMDs among triage nurses.

**Table 3:** Suspected CMDs in the sample and their association with abandonment or transfer from triage and risk classification services, Rio de Janeiro, RJ, Brazil, 2022

Variables	Suspected CMDs		*p* value*^2^*
	Cutoff point < 7	Cutoff point > 7	
	n30*^1^*	n26*^1^*	
Have you ever considered leaving your job or requesting a transfer to another department due to episodes of violence in the Risk Classification in which you were involved?			0.015
No	21 (70%)	10 (38%)	
Yes	9 (30%)	16 (62%)	
Have you ever considered leaving your job or requesting a transfer due to episodes of violence in the risk classification that you witnessed or became aware of?			0.2
No	20 (67%)	13 (50%)	
Yes	10 (33%)	13 (50%)	

Notes: Common Mental Disorders (CMDs).

1 Median (IQR);

2 Chi-Square.

Table 4 presents the results for the association between episodes of violence (threats) and suspected CMDs. Regarding the variable “threats,” the results show a statistically significant association with CMDs (p=0.031).

**Table 4:** Suspected CMDs in the sample and their association with episodes of violence (threats), Rio de Janeiro, RJ, Brazil, 2022

Variables	Suspected CMDs		*p* value*^2^*
	Cutoff point < 7	Cutoff point > 7	
	n30*^1^*	n26*^1^*	
Threat(s) in the last 12 months while performing your professional activities			0.031
No	14 (47%)	5 (19%)	
Yes	16 (53%)	21 (81%)	
The threat(s) were made by^*^			
Patient			0.4
No	4 (25%)	3 (14%)	
Yes	12 (75%)	18 (86%)	
Companion			0.5
No	3 (19%)	7 (33%)	
Yes	13 (81%)	14 (67%)	
Coworker			0.7
No	11 (69%)	16 (76%)	
Yes	5 (31%)	5 (24%)	
Which coworker			
Medical professional	4 (80%)	3 (60%)	0.2
Non-medical professional	1 (20%)	2 (40%)	

Notes: Common Mental Disorders (CMDs); N= <7 (16), > 21.

1 Median (IQR);

2 Chi-Square.

In Table 5, symptoms are grouped into four categories: depressive/anxious mood, decreased vital energy, somatic symptoms, and depressive thoughts. The table highlights the most prevalent predictive signs and symptoms of CMDs. Among the grouped symptoms identified in the SRQ-20, the most prominent complaint in Factor I (depressive/anxious mood) was the question “Do you feel nervous, tense, or worried?” with 42 positive responses (75%).

**Table 5:** Frequencies and percentages of responses to the SRQ-20 scale, Rio de Janeiro, RJ, Brazil, 2022

SRQ-20 Factor Group	Yes	No
	*f* (%)	*f* (%)
Factor 1 - Depressed/anxious mood		
Q-4 Do you get scared easily?	27 (48)	29 (52)
Q-6 Do you feel nervous, tense, or worried?	42 (75)	14 (25)
Q-9 Do you feel unhappy?	39 (70)	17 (30)
Q-10 Do you cry more than usual?	11 (20)	45 (80)
Fator 2 - Decréscimo de energia vital		
Q-8 Are you unable to think clearly?	19 (34)	37 (66)
Q-11 Do you find it difficult to enjoy your daily activities?	21 (37)	35 (63)
Q-12 Do you find it difficult to make decisions?	21 (37)	35 (63)
Q-13 Is your daily work a suffering (torment)?	13 (23)	43 (77)
Q-18 Do you feel tired all the time?	36 (64)	20 (36)
Q-20 Do you get tired easily?	39 (70)	17 (30)
Factor 3 - Somatic symptoms		
Q-1 Do you have frequent headaches?	33 (59)	23 (41)
Q-2 Do you have a lack of appetite?	13 (23)	43 (77)
Q-3 Do you sleep poorly?	41 (73)	15 (27)
Q-5 Do your hands shake?	10 (18)	46 (82)
Q-7 Do you have digestive problems?	24 (43)	32 (57)
Q-19 Do you have unpleasant sensations in your stomach?	25 (45)	31 (55)
Factor 4 - Depressive thoughts		
Q-14 Are you unable to play a useful role in life?	9 (16)	47 (84)
Q-15 Have you lost interest in things?	8 (14)	48 (86)
Q-16 Do you think you are a worthless person?	4 (7)	52 (93)
Q-17 Do you have thoughts of ending your life?	6 (11)	50 (89)

*Notes: Self Report Questionnaire-20 (SRQ-20).*

## DISCUSSION

The occupational characterization of nurses participating in this study demonstrated numerous possibilities for the emergence CMDs among professionals working in triage and risk classification settings. A notable aspect concerns the predominance of female professionals in these settings, who are more susceptible to violence and mental disorders. This finding may be linked to gender-based violence, influenced by both biological roles and sociological differences, as defined by the ILO ^(13)^.

This aligns with a study conducted in Taiwan, which revealed that female nurses experience psychological violence at a significantly higher rate than their male counterparts in emergency services, emphasizing the need for resilient measures ^(14)^. The predominantly female nursing workforce is more vulnerable to threats and is often subjected to violence, primarily perpetrated by men with authoritarian and dominating behaviors. Historically, this submission perpetuates the notion of male superiority, fostering a belief that victims owe obedience, rooted in power dynamics that promote disrespect and distortion ^(15)^.

Conversely, another significant finding is the low rate of training for professional practice in triage services among nurses. The lack of professional training impedes standardization and hinders the successful implementation of protocols in emergency and urgent care networks ^(16-18)^. This exposes professionals to vulnerabilities during patient care, resulting in anxiety and distress in their work, especially as the country’s emergency services increasingly rely on triage and risk classification as pivotal interventions for reorganizing patient flow and optimizing health promotion actions.

Evaluating satisfaction with risk classification services also relates to creating a more welcoming environment, emphasizing cleanliness, comfort, and particularly patient education regarding waiting times for medical care ^(19)^. Consequently, fostering educational actions in professional discourse is essential to align with current health policies that promote humanized care and continuous learning.

Another notable result is the significant association (p=0.015) between nurses’ intention to leave their job or request a transfer. A Brazilian study on emergency services indicates that increasing workplace violence and decreasing job satisfaction heighten the likelihood of professionals intending to leave their unit, institution, or profession. More qualified workers and those with longer tenures at an institution were more likely to consider leaving the profession ^(20)^. This aligns with current transformations in the labor market and the repercussions of the dismantling of social rights, health policies, and nursing work conditions. The influence of work conditions on professional satisfaction, illness processes, and even the desire to leave the profession is alarming, as these issues demotivate professionals and negatively impact the quality of care provided to the population ^(8,21,22)^.

Table 4 identifies patient threats as the primary source of violence experienced by nurses in triage and risk classification. A French observational study revealed that such incidents predominantly occur at night, often initiated by male patients and sometimes exacerbated by accompanying individuals. Verbal threats were the most common form of violence, followed by physical threats, with motivations related to patients’ pain, alcohol and drug use, and long waiting times. The study also noted that professionals perceived violence as a normal behavior in emergency settings ^(23)^.

The impact of violence on healthcare professionals’ mental health is well-documented. Violence contributes to fatal and non-fatal traumas, resulting in adverse consequences, including death, lost or reduced work hours due to disability, depression, burnout, and increased rates of defensive medical practices. The ultimate outcome is inferior quality of care, reduced patient satisfaction, higher frequencies of adverse events, decreased access to care, diminished team productivity, and increased worker turnover ^(24)^.

Burnout can lead to abrupt decisions by healthcare professionals, potentially harming patients. Therefore, all efforts should be made to address violence against healthcare providers and create a supportive work environment. This would enable professionals to focus on patient management rather than their safety concerns ^(25)^. A study reported improved attitudes among nurses toward aggressive behavior following participation in a prevention and management program for hostile conduct ^(25)^.

Finally, Table 5 revealed the presence of non-psychotic morbidity through emotional symptoms and issues, with notable occurrences of depressive mood (75%), sleep difficulties (73%), and depressive symptoms (84%). Emergency settings are known to be stressful environments characterized by exhausting routines, continuous care demands, exposure to traumatic situations, pain, and human suffering, necessitating high levels of emotional intelligence. When excessive and prolonged physical and emotional resource consumption occurs without adequate recovery, fatigue and stress accumulate, potentially exacerbating psychosomatic symptoms, leading to burnout, apathy, illness, or work incapacity ^(26)^.

A Brazilian study indicates that CMDs reduce productivity among nurses. It further emphasizes that the nursing profession often neglects mental health symptoms such as exhaustion, stress, and fatigue, frequently misinterpreting them as normal outcomes of demanding routines. Additionally, nurses are often self-critical and possess a strong sense of commitment, making them resistant to admitting illness or taking absences, as such actions are perceived as weaknesses, further exacerbating their vulnerability to mental disorders^(26)^.

Humor is seen as a useful form of communication for patient-centered care, as it can be used to build and maintain a relationship. Thus, it is suggested that humor improves the understanding of therapeutic concepts and leads to greater acceptance and therapeutic adherence, that is, it also results in the reduction of challenges for caregivers. The use of humor by nurses is interpreted by patients as a positive characteristic of the nurse and is also an important aspect of patient/nurse interaction^(27)^. As a result, nurses in the reception area with a risk classification who suffer from violence present mood instability, being depreciated, which reduces their enthusiasm and interest in their work.

The nurses reported that they had difficulty sleeping, corresponding to 73% of the sample. It was found that the worse the quality of sleep, the greater the perceived stress, the lower the job satisfaction, the lower the need for work shifts and the greater the chances of depressive symptoms. Depression was not far behind, with 84% of nurses in the classification responding positively to this disorder, becoming a major source of discontent related to violence in the workplace. Poorer quality of sleep and worse perceived stress are more associated with depressive symptoms, especially if nurses work more shifts and have less experience or job satisfaction^(28)^.

This data highlights the level of concern of nurses and, above all, the notion of the urgency of thinking about strategies to prevent it. In order to identify possible signs of attempted suicide and promote actions in favor of the lives of these workers, it is necessary to create spaces and moments of sensitive listening, in which feelings, needs and desires can be revealed, in addition to minimizing anguish. These spaces can provide well-being and help in resolving conflicts in the workplace.

It is crucial for nurses to remain vigilant about episodes of violence and report them when they become victims or witnesses, given the repercussions for mental, physical, and social health, which can directly affect workplace quality of life. Nurses and all healthcare workers must be encouraged to recognize and report incidents at all appropriate levels, ensuring visibility and fostering workplace discussions^(29)^.

Reporting violence through the public health system represents an essential strategy for initiating preventive actions and promoting worker health. Occupational health surveillance addresses psychosocial risks, often neglected in such initiatives. Therefore, consistent reporting is vital, as the National Occupational Health Policy emphasizes the need for strategic action planning for workers exposed to workplace violence^(30)^.

Although much has been said about interventions to minimize and protect against violence at both personal and professional levels, there is a need to go further. Greater incentives and awareness from regulatory bodies and, most importantly, attentive listening from healthcare managers are necessary to demonstrate interest and implement resolutions in such cases.

### Study Limitations

The sample size in this study serves as a limitation, as it does not allow for generalization due to its focus on emergency services in a single municipality. Additionally, the limited number of TRC professionals-one or two nurses per service-further constrained the final sample size. Another aspect refers to self-reported assessment, which may have responses influenced by conflicts of interest, generating a possibility of underestimating cases of CMD.

### Contributions to Nursing, Health, or Public Policy

Violence is a multidimensional phenomenon that can be prevented and, thus, minimized. However, effectively addressing this issue requires contributions from institutional sectors and strategic interventions with unified goals. Everyone’s involvement is needed to prevent the deterioration of workers’ mental health.

## CONCLUSIONS

This study observed no statistically significant differences in the association between violence experienced by triage nurses and CMDs regarding sociodemographic and occupational data, except for the workplace (UPA), which demonstrated a borderline association (p=0.052). Episodes of threats and the variable “intention to leave or request transfer” showed significant associations (p=0.031) and (p=0.015), respectively.

According to the data, nurses in the reception area classified as victims of violence within the workplace were suspected of having CMDs, evidenced by complaints identified by the SRQ-20, such as anxiety, depressive reactions, decreased vital energy and somatization (sadness, anxiety, fatigue, irritability, insomnia, forgetfulness, difficulty concentrating and making decisions).

CMDs and workplace violence remain pervasive realities in professional settings but are still poorly analyzed, often neglected, and under-discussed. Greater attention from managers, institutions, and workers is required to address their significant implications for various groups.
