# Supplementary material for: Violence suffered by nurses in risk classification and suspicion of common mental disorders
Source: Rev Bras Enferm. 2025 Jun 20;78(2):e20240087. doi: 10.1590/0034-7167-2024-0087 (PMC12185145; doi:10.1590/0034-7167-2024-0087)
Supplement: Translation

# Translation: Violência sofrida por enfermeiros na classificação de risco e a suspeição de transtornos mentais comuns

**DOI:** 10.1590/0034-7167-2024-0087pt

**Authors:** Daniel da Silva Granadeiro, Raquel Magalhães de Azeredo Granadeiro, Harlon França de Menezes, Renata da Silva Hanzelmann, Giovana Cópio Vieira, Juliane Ferreira da Silva, Maria Isabel Silva Santos, Joanir Pereira Passos

**Affiliations:** I Universidade do Estado do Rio de Janeiro. Rio de Janeiro, Rio de Janeiro, Brasil; II Centro Universitário Augusto Motta. Rio de Janeiro, Rio de Janeiro, Brasil; III Hospital Pró-Cardíaco. Rio de Janeiro, Rio de Janeiro, Brasil; IV Faculdade São José. Rio de Janeiro, Rio de Janeiro, Brasil; V Universidade Federal do Estado do Rio de Janeiro. Rio de Janeiro, Rio de Janeiro, Brasil

**Keywords:** Violência no Trabalho, Transtornos Mentais, Condições de Trabalho, Triagem, Enfermeiros e Enfermeiras.

## Abstract

**Objetivo:**

Analisar a associação entre a violência sofrida por enfermeiros do serviço de acolhimento com classificação de risco e a suspeição dos transtornos mentais comuns.

**Métodos:**

Estudo transversal, realizado no Município do Rio de Janeiro, com 56 enfermeiros do acolhimento com classificação de risco da rede de urgência e emergência. Utilizaram-se o Questionário para Avaliação da Violência no Trabalho e o *Self-Report Questionaire*. Foram empregados os testes de Wilcoxon, qui-quadrado, Exato de Fisher para análise.

**Resultados:**

A variável ameaça teve diferença estatística significativa (p= 0.031). Entre os sintomas agrupados, a queixa predominante do fator 1 (humor depressivo/ansioso) na questão “sente-se nervoso, tenso ou preocupado” teve 42 (75%) respostas positivas.

**Conclusão:**

A violência no acolhimento sofrida por enfermeiros reflete na suspeição de transtornos mentais, principalmente relacionada ao ambiente, abandono e sintomas depressivos.

## INTRODUÇÃO

A violência no trabalho é caracterizada como toda ação, incidente ou comportamento desviado do que é comum. Nesta situação, a pessoa é atacada, ameaçada, humilhada ou ferida por outra no exercício de suas funções^(1)^. Este fenômeno, dentro da área de saúde, mais especificamente vivenciado pelos enfermeiros, é considerado prevalente pelos órgãos internacionais de saúde, variando entre 17% e 94%, sendo citado, inclusive, que entre 8% e 38% dos profissionais sofreram violência em algum momento de sua carreira^(2,3)^.

O processo de trabalho de enfermeiros nos serviços de urgência e emergência é pautado em buscar o acolhimento, por meio da ferramenta da classificação de risco, e propor resolutividade, de forma ágil e oportuna. A classificação de risco visa avaliar e identificar as necessidades de atendimento prioritário, de acordo com a gravidade clínica, potencial de risco, agravos à saúde ou grau de sofrimento, sendo a primeira ação assistencial^(4)^. Logo, o enfermeiro que classifica deve se apoiar no raciocínio clínico por meio de métodos próprios do seu conhecimento, adotar uma classificação imediata e pautar suas ações com vistas à humanização da assistência.

Os cenários de emergência são dinâmicos, ou seja, possuem processos para a efetivação do atendimento ao paciente. Um estudo aponta que o tempo de espera entre a abertura do cadastramento e a classificação de risco pode variar de acordo com a demanda do momento, sendo possível um maior tempo de espera e demora no início do tratamento^(5)^. Desse modo, é neste período que repercussões violentas podem ocorrer.

Nesse sentido, a classificação de risco pode ser uma fase tensa, que resulta em sobrecarga e estresse, repercutindo no adoecimento do profissional de forma física ou psíquica. Os estressores do ambiente de trabalho representam potenciais determinantes para a saúde do profissional, resultando, por exemplo, em Transtornos Mentais Comuns (TMC). O TMC é caracterizado por sintomas não psicóticos, relacionados ao estresse, ansiedade e depressão, que alteram o funcionamento do organismo, levando a insônia, irritabilidade, fadiga, dificuldade de concentração, esquecimento e queixas somáticas^(6,7)^.

A presença de TMC repercute na qualidade da assistência e segurança do paciente, nas relações com a equipe de trabalho, com a família e a sociedade, na satisfação com o trabalho, com alterações na saúde do profissional e com absenteísmo, além de gerar gastos extras às instituições. A assistência à saúde pode afetar diretamente os profissionais dessa área, trazendo alterações na qualidade de vida, nos indicadores organizacionais, bem como na produção do cuidado^(8,9)^.

Com o intuito de compreender a violência no trabalho da enfermagem, seus espaços e a correlação com a saúde do trabalhador, faz-se necessário entender a relação do profissional com o trabalho e suas condições de bem-estar mental. A violência sofrida pelos enfermeiros vem sendo tema de pesquisa no Brasil e no mundo. Contudo, quando se trata de estudar esse fenômeno no serviço de acolhimento com classificação de risco e a suspeição de TMC, existem lacunas a serem preenchidas nesse tema. Para entender esse fenômeno e seu contexto, buscaram-se produções acadêmicas, mas constatou-se que a temática ainda é insuficientemente explorada.

Entender a influência da organização do trabalho na qualidade de vida, na saúde mental, na geração de sofrimento psíquico, no desgaste e no adoecimento dos trabalhadores é de fundamental importância não somente para compreensão e para intervenção em situações de trabalho que possam acarretar diversas formas de sofrimento, mas para superação e a transformação dessas organizações de trabalho.

## OBJETIVO

Analisar a associação entre a violência sofrida por enfermeiros do serviço de acolhimento com classificação de risco e a suspeição de TMC.

## MÉTODOS

### Aspectos éticos

O presente estudo foi aprovado pelo Comitê de Ética da instituição proponente e pelas demais instituições que foram incluídas como coparticipantes, atendendo aos preceitos éticos para pesquisa, de acordo com a Resolução do Conselho Nacional de Saúde nº 466/2012.

### Desenho, período e local de estudo

Estudo transversal, guiado pela ferramenta *Strengthening the Reporting of Observational Studies in Epidemiology* (STROBE), realizado na cidade do Rio de Janeiro, Brasil. Os dados foram coletados no período de outubro a dezembro de 2021, nas unidades de classificação de risco dos hospitais gerais, Coordenações de Emergência Regional (CERs) e Unidades de Pronto Atendimento (UPAs) da rede de urgência e emergência da Secretaria Municipal de Saúde (SMS).

Atualmente, a SMS conta com 29 unidades de urgência e emergência dentro de suas cinco áreas programáticas (APs). A escolha dos cenários justifica-se pelo fato de os atendimentos municipais serem os de maior cobertura na rede do Sistema Único de Saúde do estado e, portanto, setores que tendem a apresentar maior risco de ocorrências de atos violentos no ambiente de trabalho.

Os atendimentos prestados à população iniciam-se pelo acolhimento com classificação de risco. As unidades contam com instalações que dão suporte ao tratamento e recuperação dos usuários vítimas de agravos clínicos e traumáticos, possuindo unidades de internação, cirurgia e tratamento intensivo.

### População, critérios de inclusão e exclusão

A população foi composta por enfermeiros que atuavam na classificação de risco das unidades. A amostra foi selecionada de forma não probabilística, por meio da estratégia de recrutamento consecutivo *Snowball*, para alcançar os participantes mediante cadeias de referência^(10)^.

Os critérios de inclusão foram: enfermeiros em pleno exercício no período da coleta de dados, independentemente do vínculo empregatício em suas respectivas unidades de urgência e emergência; que exercessem suas atividades laborativas por um período mínimo de 12 meses no serviço de acolhimento; e que possuíssem e-mail ou o aplicativo móvel WhatsApp® instalado em seu dispositivo telefônico. A carga horária desses profissionais é de 30 horas semanais, cumpridas em regime de turnos (diurno e noturno).

O tempo de serviço foi determinado por se entender que esse período é essencial no que diz respeito às vivências e às experiências relacionadas ao ambiente de trabalho e pela padronização do tempo mínimo exigido para utilização da escala. Por sua vez, foram excluídos enfermeiros sem vínculo trabalhista com os serviços (estagiários, residentes, preceptores e acadêmicos), profissionais ausentes por motivos de férias, licença-saúde, licença-gestação e/ou atestado médico e os questionários que apresentaram menos que 50% de respostas preenchidas.

### Protocolo do estudo

Para a coleta de dados, inicialmente foram utilizadas cinco cadeias de referências, neste caso, as unidades hospitalares que integram cada Área Programática (AP). Devido às ações vigentes pelos órgãos oficiais de saúde para o enfrentamento da COVID-19 à época, a coleta de dados foi feita por meio eletrônico.

Após contato com os profissionais de cada unidade, foi realizado o agendamento prévio para solicitação do endereço eletrônico ou contato telefônico vinculado ao aplicativo WhatsApp® para o envio do endereço (link) de acesso aos instrumentos de coleta de dados da pesquisa, por meio do Google *Forms*®. O *layout* do Google *Form*s® foi dividido em quatro blocos distintos: Termo de Consentimento Livre e Esclarecido (TCLE), Questionário Sociodemográfico, Questionário para Avaliação da Violência no Trabalho e o *Self-Report Questionnaire* (SRQ-20).

Logo após o período de coleta de dados, o link foi excluído e os dados coletados foram importados do Google *Forms*® e armazenados em uma pasta local por meio do programa Microsoft Excel®. O TCLE online foi a página inicial do formulário, onde estavam as informações sobre a pesquisa e a solicitação de autorização para o uso dos dados. O participante só teve acesso aos questionários para autopreenchimento após o aceite eletrônico do TCLE. O Questionário Sociodemográfico era composto por 21 perguntas, abertas e fechadas, com o objetivo de caracterizar os participantes da pesquisa.

O Questionário para Avaliação da Violência no Trabalho (Survey of Violence in the Workplace for Health Care Workers) foi adaptado e elaborado pela *American Federation of State, County and Municipal Employees* ^(11)^. O questionário foi estruturado em seções, tendo como base o *Survey Questionnaire Workplace Violence in the Health Sector*, proposto pela Organização Mundial da Saúde (OMS) e pela Organização Internacional do Trabalho (OIT).

Para obtenção dos dados, foram feitas adequações e adaptações visando atender às peculiaridades do acolhimento com classificação de risco e da população pesquisada (enfermeiros), pois o instrumento original era destinado a todos os setores e profissionais da saúde. O questionário resultante foi estruturado em cinco seções, com 46 questões, assim distribuídas: condições de segurança do local de trabalho (três questões), treinamento em violência no trabalho (duas questões), políticas de prevenção e abordagem da violência no trabalho (cinco questões), episódios de violência no trabalho (29 questões) e opinião pessoal (oito questões).

O *Self-Report Questionnaire* (SRQ) é recomendado pela OMS e validado por pesquisadores brasileiros desde 1986. É composto por 20 questões de fácil aplicação, com respostas “sim” e “não”, sobre a presença de sintomas físicos e psíquicos nos últimos 30 dias. São avaliados sintomas psicossomáticos, como dores de cabeça, má digestão e sensações desagradáveis no estômago; sintomas depressivos, como tristeza, choro frequente e perda de interesse; e sintomas ansiosos, como dormir mal, assustar-se com facilidade, tremores e nervosismo^(9)^.

O instrumento desempenha um papel relevante na avaliação dos TMC, demonstrando habilidade para identificar fatores que, em conjunto, denotam características indispensáveis para o rastreamento da saúde mental em âmbito ocupacional. Para definir a suspeição de TMC, o ponto de corte utilizado no estudo foi 7 para ambos os sexos. Este ponto foi estabelecido, no estudo de validade do questionário, como o mais adequado em termos de sensibilidade e especificidade ^(12)^.

### Análise dos resultados e estatística

A análise dos dados foi realizada por meio de estatística descritiva, apresentada em tabelas, com frequências absolutas (n) e relativas (%), média, desvio padrão, mediana, valores máximos e mínimos da frequência percentual simples, média aritmética e desvio padrão.

A análise descritiva foi realizada utilizando medidas de tendência central e distribuição de frequências relativas de cada variável qualitativa dicotômica entre os suspeitos ou não de TMC, estimando a prevalência em cada situação e avaliando a significância da associação. A análise dos fatores de risco para a ocorrência ou não de TMC considerou um modelo de regressão logística binária, tendo como variáveis os preditores sociodemográficos, ocupacionais e de violência.

A análise inferencial foi realizada utilizando o teste de Wilcoxon para comparações entre o desfecho e variáveis numéricas. Para comparações com variáveis categóricas, foi utilizado o teste qui-quadrado quando todos os substratos da tabela de contingência eram maiores que 5, e o teste exato de Fisher para variáveis com cinco observações ou menos em algum substrato. O nível de significância adotado foi de 5%. O software utilizado foi o R, versão 4.0.

## RESULTADOS

A amostra final foi composta por 56 enfermeiros, com predominância do sexo feminino (n = 41/73%), brancos (n = 25/45%), solteiros (n = 32/57%) e com idade entre 34 e 45 anos. No que diz respeito à escolaridade, 48 (86%) participantes possuíam especialização, e 33 (59%) enfermeiros não haviam recebido nenhuma capacitação para desenvolver sua prática profissional no serviço.

Na Tabela 1 é apresentada a distribuição das características ocupacionais entre os enfermeiros do acolhimento com classificação de risco.

**Tabela 1:** Distribuição dos participantes do estudo segundo as características ocupacionais, Rio de Janeiro, RJ, Brasil, 2022

Variáveis	*F*	%
Área da cidade do Rio de Janeiro em que trabalha		
Centro	9	16
Zona Norte	23	41
Zona Oeste	16	29
Zona Sul	5	9
Não sei informar	3	5
Local de trabalho^*^		
UPA	31	55
CER	10	18
Hospital Geral	19	34
Recebeu capacitação para trabalhar no ACCR		
Não	33	59
Sim	23	41
Turno de atuação no ACCR		
Serviço Diurno	12	21
Serviço Noturno	4	7
Ambos	40	72
Contrato de Trabalho^*^		
Estatutário	5	9
CLT	39	70
Terceirizado (OSS)	17	30
Total de horas de trabalho no ACCR		
Maior que 30 horas	17	30
Menor ou = 30 horas	39	70
Número de vínculos empregatícios		
1	24	43
2 ou mais	32	57
Conhecimento sobre violência no ambiente de trabalho		
Não	1	2
Sim	54	96
Não sei informar	1	2

Legenda:

* Nesta variável o participante pôde marcar mais de uma opção; UPA (Unidade de Pronto Atendimento); CER (Coordenação de Emergência Regional); ACCR (Acolhimento com Classificação de Risco); CLT (Consolidação das Leis do Trabalho); OSS (Organizações Sociais de Saúde).

Quanto às variáveis relacionadas à área da cidade do Rio de Janeiro onde trabalham, capacitação para atuar no acolhimento, tempo de atuação, turno e horas de trabalho, número de vínculos empregatícios, total de horas semanais de trabalho e tempo de exercício profissional, não foi encontrada diferença estatística significativa (p < 0,05) para a associação com a suspeição de TMC (Tabela 2).

**Tabela 2:** Associação entre características ocupacionais e suspeição de Transtornos Mentais Comuns, Rio de Janeiro, RJ, Brasil, 2022

Variáveis	Suspeição de TMC		*p* value*^2^*
	Ponto de corte < 7	Ponto de corte > 7	
	n30*^1^*	n26*^1^*	
Local de Trabalho			
UPA			
Não	17 (57%)	8 (31%)	0,052
Sim	13 (43%)	18 (69%)	
CER			
Não	24 (80%)	22 (85%)	0,7
Sim	6 (20%)	4 (15%)	
Hospital Geral			
Não	17 (57%)	20 (77%)	0,11
Sim	13 (43%)	6 (23%)	

*Legenda: Transtornos Mentais Comuns (TMC); Unidade de Pronto Atendimento (UPA); Coordenação de Emergência Regional (CER).*

Nota:

1 Mediana (IQR);

2 Qui-quadrado.

A Tabela 3 apresenta as associações das variáveis abandono e/ou transferência do acolhimento com classificação de risco, devido à vivência ou testemunho de episódios de violência, com os TMC entre os enfermeiros classificadores.

**Tabela 3:** Associação entre abandono ou transferência por episódios de violência e suspeição de Transtornos Mentais Comuns, Rio de Janeiro, RJ, Brasil, 2022

Variáveis	Suspeição de TMC		*p* value*^2^*
	Ponto de corte < 7	Ponto de corte > 7	
	n30*^1^*	n26*^1^*	
Você já pensou em abandonar o emprego ou solicitar transferência de setor em virtude de episódios de violência na Classificação de Risco em que você esteve envolvido?			0,015
Não	21 (70%)	10 (38%)	
Sim	9 (30%)	16 (62%)	
Você já pensou em abandonar o emprego ou solicitar transferência em virtude de episódios de violência na Classificação de Risco que você testemunhou ou tomou conhecimento?			0,2
Não	20 (67%)	13 (50%)	
Sim	10 (33%)	13 (50%)	

Legenda: Transtornos Mentais Comuns (TMC). Nota:

1 Mediana (IQR);

2 Qui-Quadrado.

A Tabela 4 mostra os resultados obtidos para a associação entre episódios de violência (ameaça) e a suspeição de TMC. No que diz respeito à variável ameaça, os resultados apresentados indicam uma associação significativa com os TMC, evidenciada pela diferença estatística (p = 0,031).

**Tabela 4:** Suspeição de Transtornos Mentais Comuns na amostra e associação com episódios de violência (ameaça), Rio de Janeiro, RJ, Brasil, 2022

Variáveis	Suspeição de TMC		*p* value*^2^*
	Ponto de corte < 7	Ponto de corte > 7	
	n30*^1^*	n26*^1^*	
Ameaça nos últimos 12 meses exercendo suas atividades profissionais			0,031
Não	14 (47%)	5 (19%)	
Sim	16 (53%)	21 (81%)	
A(s) ameaça(s) foram feitas por^*^			
Paciente			0,4
Não	4 (25%)	3 (14%)	
Sim	12 (75%)	18 (86%)	
Acompanhante			0,5
Não	3 (19%)	7 (33%)	
Sim	13 (81%)	14 (67%)	
Colega de trabalho			0,7
Não	11 (69%)	16 (76%)	
Sim	5 (31%)	5 (24%)	
Qual colega de trabalho			
Profissional médico	4 (80%)	3 (60%)	0,2
Profissional não médico	1 (20%)	2 (40%)	

Legenda: Transtornos Mentais Comuns (TMC); N = <7 (16), >21. Nota:

1 Mediana (IQR);

2 Qui-quadrado.

Na Tabela 5, os sintomas são divididos em quatro grupos (humor depressivo/ansioso, decréscimo de energia vital, sintomas somáticos e pensamentos depressivos), sendo apresentadas as respostas positivas dos sinais e sintomas preditores mais prevalentes de TMC. Entre os sintomas agrupados presentes no SRQ-20, a queixa predominante do fator I (humor depressivo/ansioso), referente à questão “Sente-se nervoso, tenso ou preocupado”, teve 42 (75%) respostas positivas.

**Tabela 5:** Frequências e porcentagens das respostas à escala SRQ-20, Rio de Janeiro, RJ, Brasil, 2022

Grupo de Fatores do SRQ-20	Sim	Não
	*f* (%)	*f* (%)
Fator 1 - Humor depressivo/ansioso		
Q-4 Fica com medo com facilidade?	27 (48)	29 (52)
Q-6 Sente-se nervoso(a), tenso(a) ou preocupado(a)?	42 (75)	14 (25)
Q-9 Sente-se infeliz?	39 (70)	17 (30)
Q-10 Chora mais que o comum?	11 (20)	45 (80)
Fator 2 - Decréscimo de energia vital		
Q-8 Não consegue pensar com clareza?	19 (34)	37 (66)
Q-11 Acha difícil gostar de suas atividades diárias?	21 (37)	35 (63)
Q-12 Acha difícil tomar decisões?	21 (37)	35 (63)
Q-13 Seu trabalho diário é um sofrimento (tormento)?	13 (23)	43 (77)
Q-18 Sente-se cansado(a) o tempo todo?	36 (64)	20 (36)
Q-20 Fica cansado(a) com facilidade?	39 (70)	17 (30)
Fator 3 - Sintomas somáticos		
Q-1 Você tem dores de cabeça frequentes?	33 (59)	23 (41)
Q-2 Tem falta de apetite?	13 (23)	43 (77)
Q-3 Dorme mal?	41 (73)	15 (27)
Q-5 Suas mãos tremem?	10 (18)	46 (82)
Q-7 Tem problema digestivo?	24 (43)	32 (57)
Q-19 Tem sensações desagradáveis no estômago?	25 (45)	31 (55)
Fator 4 - Pensamentos depressivos		
Q-14 Não é capaz de ter um papel útil na vida?	9 (16)	47 (84)
Q-15 Perdeu o interesse pelas coisas?	8 (14)	48 (86)
Q-16 Acha que é uma pessoa que não vale nada?	4 (7)	52 (93)
Q-17 Tem o pensamento de acabar com a vida?	6 (11)	50 (89)

*Legenda: Self Report Questionnaire-20 (SRQ-20).*

## DISCUSSÃO

A caracterização ocupacional dos enfermeiros participantes deste estudo demonstrou que são inúmeras as possibilidades para o surgimento de TMC nesses profissionais que atuam no acolhimento com classificação de risco. Um aspecto de destaque diz respeito ao maior número de profissionais do sexo feminino nos cenários de acolhimento, sendo essas profissionais mais suscetíveis à violência e aos transtornos mentais. Tal dado pode estar atrelado à violência de gênero, seja por funções e características biológicas, seja por diferenças sociológicas, conforme define a OIT^(13)^.

Isso vai ao encontro de um estudo em Taiwan que revela que as enfermeiras sofrem violência mental em uma extensão muito maior do que os homens nos serviços emergenciais, sendo necessário traçar medidas resilientes^(14)^. A equipe de enfermagem, predominantemente feminina, torna-se mais vulnerável às ameaças e, por isso, pode sofrer violência, majoritariamente, por homens, com conceitos autoritários e de dominação. Historicamente, tal submissão ainda traz a ideia de superioridade masculina, baseada na crença de que as vítimas lhes devem obediência, pautada em relações de poder social, nas quais predominam o desrespeito e o desvirtuamento^(15)^.

Por outro lado, um dado que merece atenção é a baixa capacitação para o desenvolvimento da prática profissional no serviço de acolhimento pelos enfermeiros. A falta de treinamento profissional impede uma padronização e causa dificuldade para o alcance do êxito em protocolos de qualquer natureza na rede de atenção às urgências e emergências^(16-18)^. Com isso, os profissionais se expõem a fragilidades durante o atendimento, gerando ansiedade e angústia em seu trabalho, já que os serviços de urgência e emergência do país têm investido no acolhimento com classificação de risco como uma intervenção impactante na reorganização do fluxo de atendimento na rede de saúde e na otimização das ações de promoção à saúde.

Estudo que avaliou as ações de educação permanente em saúde durante a pandemia da COVID-19 conclui que há lacunas nas ações com foco na promoção e proteção à saúde mental e no monitoramento clínico dos profissionais de saúde, evidenciando a necessidade de articulação de trabalhadores, sistemas, serviços de saúde e secretarias estaduais, de modo a acompanhar os profissionais da saúde em suas diversas categorias e locais de trabalho^(17)^. Desta forma, intervenções educativas devem ser implementadas a fim de aprimorar os conhecimentos técnicos e assistenciais dos enfermeiros na classificação de risco, proporcionando espaço para reflexão coletiva e análise da realidade em que estão inseridos e sobre a prevenção de TMCs^(16)^.

Como também visto, os cenários das UPAs são os que possuem maior número de profissionais que laboram neste ambiente, apresentando-se com associação limítrofe (p=0,052) para suspeição de TMCs. Estas instituições são potenciais para maiores fragilidades organizacionais e que geram desconfortos e, consequentemente, para a incitação de violência. A realidade destes locais pode estar atrelada a entraves, tais como má comunicação com a rede de saúde, superlotação dos hospitais, regulação prejudicada e transporte acessível de pacientes inadequado, e a facilidades, mediadas pela boa relação e empatia^(18)^.

Sendo assim, avaliar a satisfação do atendimento na classificação de risco também está relacionado a uma ambiência mais acolhedora, direcionada à limpeza e ao conforto, e, principalmente, à orientação ao usuário acerca do tempo de espera para o atendimento médico^(19)^. Deste modo, é imprescindível a ação educativa nos discursos dos profissionais, atendendo às prerrogativas das políticas de saúde vigentes, que incentivam a humanização da assistência e o aprendizado constante.

Outro resultado revela que a intenção de abandonar o emprego ou solicitar transferência teve associação significativa (p=0,015) entre os enfermeiros. Estudo realizado em emergências no Brasil aponta que os fatores para violência no trabalho aumentaram e a satisfação com o emprego atual diminuiu a probabilidade de maior intenção de deixar a unidade, a instituição e a profissão. Os trabalhadores mais qualificados e aqueles que trabalhavam na instituição havia mais tempo estavam mais propensos a manifestar maior intenção de deixar a profissão^(20)^. Este aspecto está em consonância com as atuais transformações no mundo do trabalho e a repercussão do desmonte dos direitos sociais, das políticas de saúde e do trabalho na enfermagem. A influência que as condições de trabalho exercem sobre a satisfação profissional, os processos de adoecimento e até mesmo o desejo de abandonar a profissão já é preocupante, pois essas questões desmotivam os profissionais e provocam efeitos negativos na qualidade da assistência prestada à população^(8,21,22)^.

A Tabela 4 apresenta a ameaça dos pacientes como principal fonte de violência sofrida pelos enfermeiros do acolhimento com classificação de risco. Um estudo observacional francês revela que as ocorrências podem acontecer mais à noite, originadas por homens, geralmente perpetradas por pacientes, mas incitadas por acompanhantes. A forma de ameaça mais comum foi a verbal, seguida da física, com motivação relacionada à dor do paciente, ao uso de álcool e drogas e aos longos tempos de espera. O estudo também revela que os profissionais consideravam a violência um comportamento normal nos cenários de emergência^(23)^.

É notória a repercussão da violência na saúde mental dos profissionais de saúde. A violência contribui para traumas fatais e não fatais, resultando em consequências negativas, como morte, horas de trabalho perdidas ou reduzidas por incapacidade, depressão, esgotamento e aumento das taxas de prática médica defensiva. O resultado final é uma assistência de qualidade inferior, diminuição da satisfação dos pacientes, aumento da frequência de eventos adversos, redução do acesso aos cuidados, menor produtividade da equipe de saúde e maior rotatividade dos trabalhadores^(24)^.

O esgotamento pode levar a decisões abruptas por parte dos profissionais de saúde, que podem até ser prejudiciais para os pacientes. Portanto, devem ser feitos todos os esforços para resolver a questão da violência contra os profissionais de saúde e proporcionar um ambiente seguro. Isso permitirá que se concentrem melhor na gestão dos pacientes, em vez de se preocuparem com sua própria segurança^(25)^. Um estudo aponta que houve melhoria nas atitudes dos enfermeiros em relação ao comportamento agressivo após a participação em um programa desenvolvido e aplicado para prevenção e gestão de comportamento hostil^(25)^.

Por fim, a Tabela 5 demonstrou a existência de morbidade não psicótica por meio de sintomas e problemas emocionais, com destaque para o humor (75%), as dificuldades relacionadas ao sono (73%) e os sintomas depressivos (84%). Como se sabe, as emergências são caracterizadas como ambientes estressantes, com rotinas desgastantes, cuidados ininterruptos e convívio com situações traumáticas, dor e sofrimento humano, o que exige extrema inteligência emocional. Quando há um consumo excessivo e prolongado de recursos físicos e emocionais ainda não totalmente recuperados, ocorre acúmulo de fadiga e estresse, potencializando sintomas psicossomáticos e resultando em esgotamento, apatia, adoecimento ou incapacidade laboral^(26)^.

Um estudo brasileiro aponta que os TMC causam perda de produtividade nos enfermeiros. O estudo ainda refere que a enfermagem tende a ignorar sintomas de adoecimento mental, como exaustão, estresse e fadiga, frequentemente confundidos com cansaço decorrente de rotinas exaustivas. Ademais, os profissionais se consideram autocríticos e possuem um senso de compromisso que os torna resistentes a adoecer ou se ausentar, o que é frequentemente compreendido como sinal de fraqueza, agravando a vulnerabilidade aos transtornos mentais^(26-28)^.

O humor é visto como forma de comunicação útil para o cuidado centrado no paciente, já que pode ser usado para construir e manter um relacionamento. Desta forma, sugere-se que o humor melhore a compreensão dos conceitos terapêuticos e leve a maior aceitação e adesão terapêutica, ou seja, resulta ainda na redução dos desafios para os prestadores de cuidados. O uso do humor pelos enfermeiros é interpretado pelos pacientes como uma característica positiva do enfermeiro e também é um aspecto importante da interação paciente/enfermeiro^(27)^. Com isso, os enfermeiros do acolhimento com classificação de risco que sofrem violência apresentam instabilidade do humor, sendo depreciados, o que diminui o ânimo e interesse no seu trabalho.

Os enfermeiros pontuaram que possuíam dificuldade para dormir, correspondendo a 73% da amostra. Foi constatado que, quanto pior a qualidade do sono, maior o estresse percebido, menor a satisfação no trabalho, menor a necessidade de turnos de trabalho e maiores as chances de sintomas depressivos. A depressão não ficou tão longe, com 84% dos enfermeiros da classificação com respostas positivas para tal transtorno, se tornando uma principal fonte de descontentamento relacionada à violência no ambiente de trabalho. A menor qualidade do sono e o pior estresse percebidos estão mais associados aos sintomas depressivos, especialmente, se os enfermeiros trabalharem mais por turnos e tiverem menos experiência ou satisfação profissional^(28)^.

Esse dado traz à luz o nível de inquietação dos enfermeiros e, principalmente, a noção sobre a urgência de se pensar em estratégias para evitá-lo. Para que possíveis sinais de tentativa de suicídio sejam identificados e promover a ações a favor da vida desses trabalhadores, é necessária a criação de espaços e momentos de escuta sensível, em que sentimentos sejam revelados, suas necessidades e seus desejos, além de minimizar a angústia. Esses espaços podem proporcionar bem-estar e auxiliar na resolução de conflitos no ambiente de trabalho.

Ressalta-se que os enfermeiros devem estar atentos aos episódios de violência sofridos e denunciá-los quando forem vítimas ou testemunhas desses atos, pois há repercussões para a saúde mental, física e social, que podem afetar diretamente a qualidade de vida no trabalho. É necessário que os enfermeiros e todos os trabalhadores de saúde tenham atitudes proativas e sejam incentivados a denunciar essas situações em todas as instâncias cabíveis, para que o fenômeno tenha maior visibilidade e seja tratado e discutido nos espaços de trabalho^(29)^.

A notificação de violência relacionada ao trabalho na rede de saúde pública é uma importante estratégia para o desencadeamento de ações de prevenção e promoção à saúde do trabalhador. Nas ações de vigilância em saúde do trabalhador, são abordados os riscos psicossociais, geralmente negligenciados nas iniciativas de vigilância. Sendo assim, é fundamental que notificações sejam feitas, considerando que a Política Nacional de Saúde do Trabalhador e da Trabalhadora prevê o replanejamento de ações para este grupo por meio do enfrentamento das violências no trabalho^(30)^.

No entanto, muito se fala sobre medidas de intervenção para minimizar e proteger contra a violência, seja em nível pessoal ou profissional. Iniciativas de reabilitação biológica, psicológica e social são bem-vindas e citadas na literatura. Contudo, é preciso ir além, incentivando a sensibilização dos órgãos reguladores e, principalmente, a escuta dos gestores de saúde, de modo a demonstrar interesse e buscar soluções para esses casos.

### Limitações do Estudo

A amostra deste estudo é um limitador, pois não permite generalizações, considerando que foi realizado nos serviços de emergência de um único município do estado. Além disso, o quantitativo de profissionais do ACCR é limitado a um ou dois enfermeiros por serviço, o que também restringe a amostra final. Outro aspecto se refere à avaliação autorreferida, que pode ter respostas influenciadas por conflitos de interesses, gerando uma possibilidade de subestimação de casos de TMC.

### Contribuições para as áreas da enfermagem, saúde, ou políticas públicas

A violência é um fenômeno multidimensional, que pode ser prevenida e, portanto, minimizada. Contudo, evitá-la de forma eficaz depende da contribuição dos setores institucionais envolvidos e de intervenções estratégicas com um objetivo comum. É preciso envolvimento de todos a fim de evitar a deterioração da saúde mental do trabalhador.

## CONCLUSÕES

Neste estudo, observou-se que, na associação entre a violência sofrida pelos enfermeiros do acolhimento com classificação de risco e os TMC, não houve diferença estatística significativa para os dados sociodemográficos e ocupacionais, salvo o local de trabalho (UPA), que apresentou associação limítrofe (p=0,052). Quanto à suspeição devido aos episódios de ameaça e à variável “abandonar ou pedir transferência do serviço”, verificou-se associação significativa (p=0.031) e (p=0,015), respectivamente.

Conforme os dados, os enfermeiros do acolhimento com classificação de risco vítimas de violência dentro do espaço de trabalho tiveram suspeição para TMCs, evidenciada através de queixas identificadas pelo SRQ-20, como a ansiedade, reações depressivas, diminuição da energia vital e somatização (tristeza, ansiedade, fadiga, irritabilidade, insônia, esquecimento, dificuldade na concentração e tomada de decisões).

Os TMC e a violência no trabalho são uma realidade presente no meio social laboral, contudo, ainda são pouco analisados, frequentemente negligenciados e pouco discutidos, apesar de sua relevância para os diferentes grupos de interesse, como gestores, instituições e trabalhadores.
